# Supplementary material for: Design of Nanostructured Hybrid Electrodes Based on a Liquid Crystalline Zn(II) Coordination Complex-Carbon Nanotubes Composition for the Specific Electrochemical Sensing of Uric Acid
Source: Nanomaterials (Basel). 2022 Nov 27;12(23):4215. doi: 10.3390/nano12234215 (PMC9739524; doi:10.3390/nano12234215)
Supplement: Supplementary file 1 [file nanomaterials-12-04215-s001.zip › nanomaterials-2051695-supplementary.pdf]

Supplementary Materials

Design of Nanostructured Hybrid Electrodes Based on a Liquid Crystalline Zn(II) Coordination Complex-Carbon Nanotubes Composition for the Specific Electrochemical Sensing of Uric Acid

Sorina Negrea [1,2], Adelina A. Andelescu [3], Sorina Ilies (b. Motoc) [3], Carmen Cretu [3], Liliana Cseh [3], Mircea Rastei [4], Bertrand Donnio [4], Elisabeta I. Szerb [3,*] and Florica Manea [1,*]

[1] Department of Applied Chemistry and Engineering of Inorganic Compounds and Environment, Politehnica University of Timisoara, Bvd. Vasile Parvan No. 6, 300223 Timisoara, Romania
[2] National Institute of Research and Development for Industrial Ecology (INCD ECOIND), Timisoara Branch, 300431 Timisoara, Romania
[3] "Coriolan Drăgulescu" Institute of Chemistry, Romanian Academy, 24 Mihai Viteazu Bvd., 300223 Timisoara, Romania;
[4] Institut de Physique et Chimie des Matériaux de Strasbourg (IPCMS), CNRS-Université de Strasbourg (UMR7504), 67034 Strasbourg, France

* Correspondence: eszerb@acad-icht.tm.edu.ro (E.I.S.); florica.manea@upt.ro (F.M.)

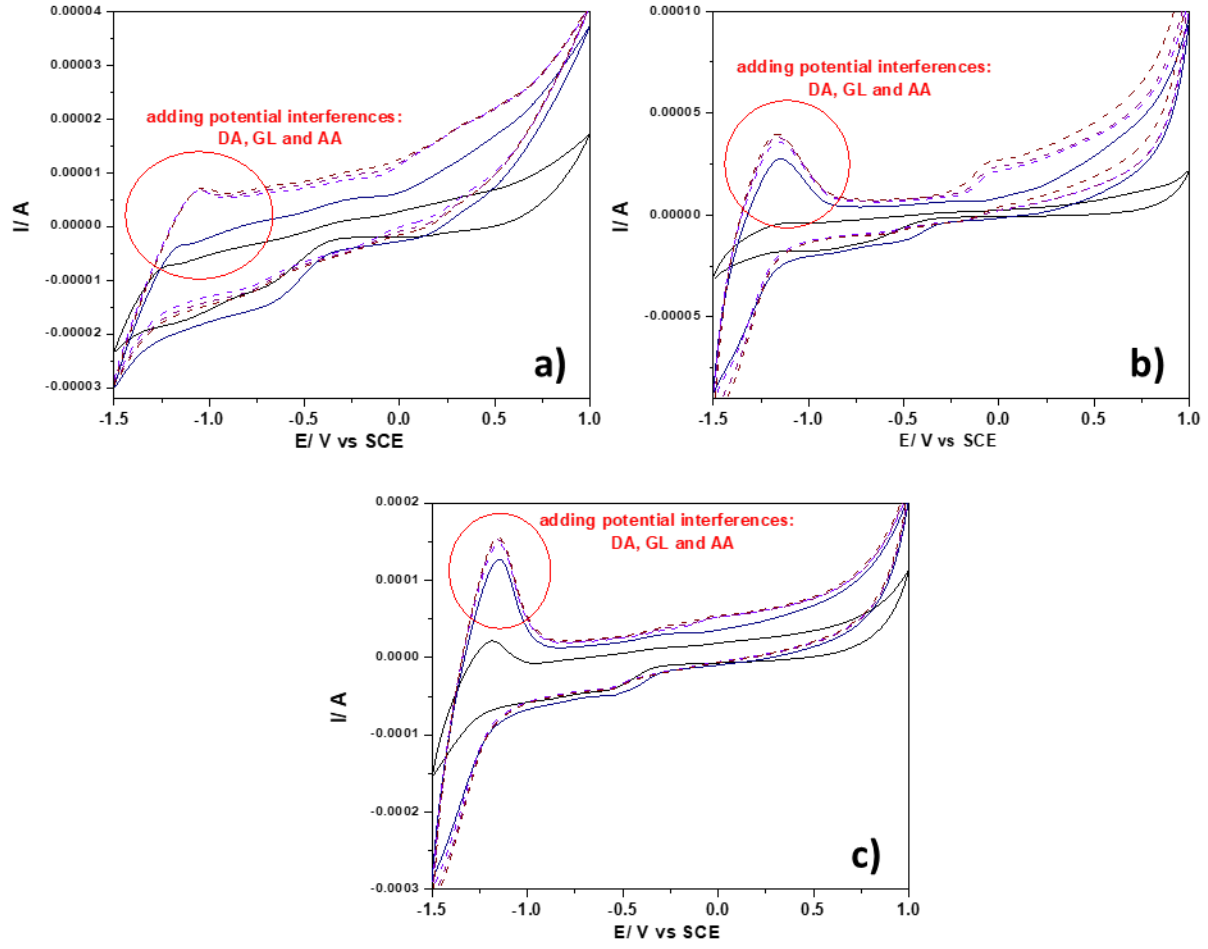

Figure S1. Cyclic voltammograms recorded at: (**a**) **PE_01** paste electrode, (**b**) **PE_02** paste electrode and (**c**) **PE_03** paste electrode, in 0.1 M NaOH supporting electrolyte (curve 1) and in the presence

of 5 mM UA concentration and potential interferences (1 mM): DA, GL and AA; potential scan rate: 0.05 V·s^{-1}; potential range from −1.5 V to +1V/Ag/ AgCl.

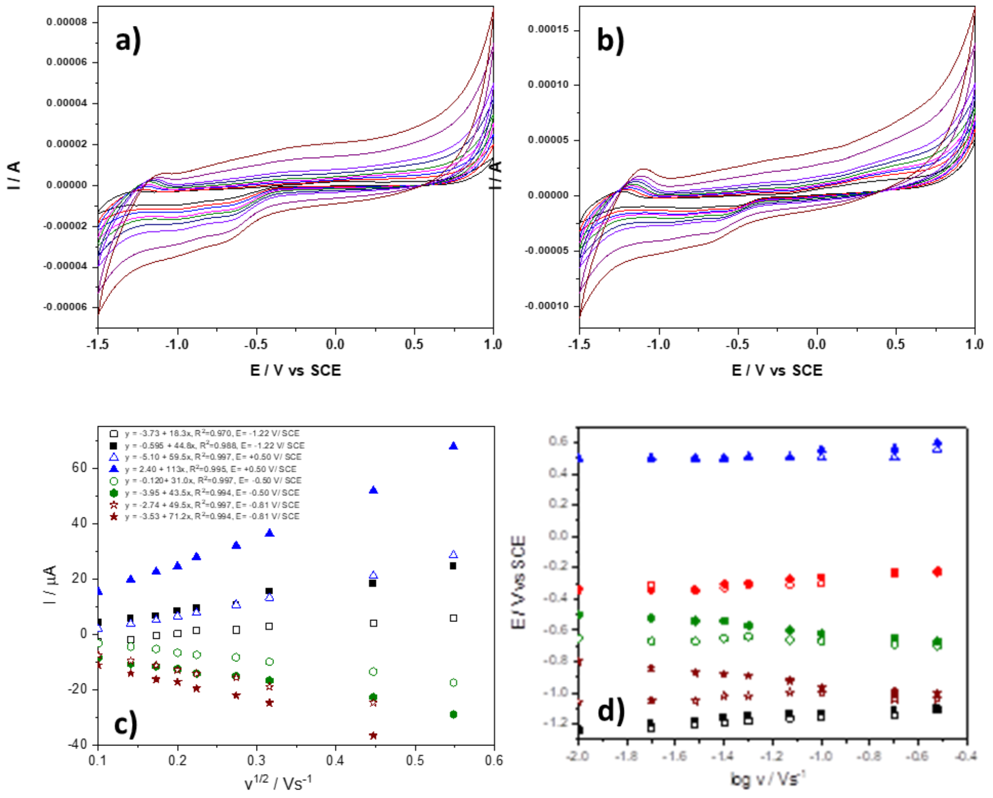

Figure S2. Cyclic voltammograms recorded in: (**a**) the absence of UA 0.1 M NaOH supporting electrolyte, and in (**b**) the presence of 3 mM UA with the **PE_03** paste electrode at various scan rates: (curve 1) 10, (curve 2) 20, (curve 3) 30, (curve 4) 40, (curve 5) 50, (curve 6) 75, (curve 7) 100, (curve 8) 200, and (curve 9) 300 mV·s^{-1}; (c) Dependence of anodic and cathodic peaks current vs. square root of the scan rate. (**d**) Dependence of peak potential vs logarithm of the scan rate.

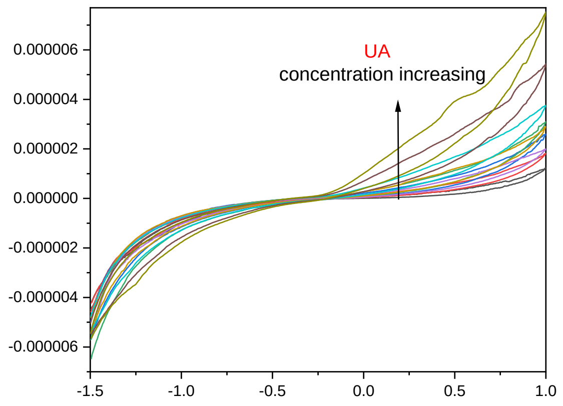

Figure S3. Cyclic voltammograms recorded at 80°C for **PE_03** paste electrode, in 0.1 M NaOH supporting electrolyte (curve 1) and in the presence of 1-8 mM UA concentrations; potential scan rate: 0.05 V·s^{-1}; potential range from −1.5 V to +1V/ SCE.
